# Magnetoelastic Humidity Sensors with TiO_2_ Nanotube Sensing Layers

**DOI:** 10.3390/s20020425

**Published:** 2020-01-11

**Authors:** Selcuk Atalay, Tekin Izgi, Veli Serkan Kolat, Sema Erdemoglu, Orhan Orcun Inan

**Affiliations:** 1 Physics Department, Faculty of Science, Inonu University, Malatya 44280, Turkey; tekin.izgi@inonu.edu.tr (T.I.); veli.kolat@inonu.edu.tr (V.S.K.); o.orcun.inan@hotmail.com (O.O.I.); 2 Chemistry Department, Faculty of Science, Inonu University, Malatya 44280, Turkey; sema.erdemoglu@inonu.edu.tr

**Keywords:** magnetoelastic sensor, humidity, TiO_2_

## Abstract

In this study, TiO_2_ nanotubes (TiO_2_-NTs) are coated with a drop-casting method on Fe_40_Ni_38_Mo_4_B_18_ amorphous ferromagnetic ribbons and the humidity response of the prepared magnetoelastic sensors (MES) is investigated. The synthesis of TiO_2_-NTs is performed using a hydrothermal process. Sample characterization is carried out using X-ray diffraction and scanning electron microscopy. The results show that the sensors can measure moisture values in the range of 5% to 95% with very high precision and very low hysteresis. The humidity variation between 5% and 95% shows a change in the sensor resonance frequency of ~3180 Hz, which is a significant change compared to many magnetoelastic humidity sensors developed so far.

## 1. Introduction

Ferromagnetic amorphous alloys containing Fe, Ni, and/or Co are excellent magnetoelastic materials. Magnetoelastic materials change their shape when exposed to a magnetic field and, conversely, undergo magnetization changes when a mechanical stress is applied. This bidirectional coupling provides the transduction capability when working as sensing devices [1,2,3,4]. It follows that when an AC magnetic field is applied to a magnetoelastic material, the sample starts to vibrate at the frequency of the applied AC magnetic field. The mechanical vibration of the magnetoelastic material is then generated by sending a time-varying magnetic signal and the magnetoelastic material would in response generate a time varying magnetic flux that could be detected with a set of pickup coils. The flux measured by pickup coils reaches a maximum at the resonant frequency of the sample. The resonant frequency depends on the oscillating mass of the sensor and the adhered layers. Therefore, if there is a change in the mass of the system, both the resonant frequency and its amplitude will be modified proportionally. The resonance frequency shift, Δ*f*, with the mass or density of adsorbed molecules by the surface of the magnetoelastic sensor is given by [4,5,6,7]:

(1) $$\Delta f=-\frac{f}{2}\frac{\Delta m}{M}$$

where Δ*m* is the variation of sensor mass and *M* is the mass of the MES before any absorption.

Magnetoelastic sensor platforms have been used for chemical and physical detection, including liquid density and viscosity [8,9,10,11,12,13,14], pH [15,16], humidity and temperature [17,18,19,20,21], and mass [22,23]. Recently, the detection of chemical gas sensors and biosensors have been developed [24,25,26,27,28,29,30,31,32,33,34]. For these kinds of detection, the immobilization of a selective and specific functional layer covering the magnetoelastic platform for binding of a target analyte has been found necessary. Several sensors have been reported for different molecule detection [31,32,33,34]. In the case of biological detection, research is usually focused on bacteria detection, for example, *E. coli* [32] and *Salmonella enterica* typhimurium [33,34].

Jain [19] developed a magnetoacoustic humidity sensor by dip-coating of an alumina sol-gel solution into an amorphous ribbon. Grimes et al. [17,18] also fabricated a humidity sensor using 2826 MB amorphous alloys. As stated above, in magnetoelastic sensors, the amount of mass usually deposited on the sensor surface causes a shift in the resonance frequency and the shift in the magnetoelastic measurement system is measured. In this way, the mass accumulated on the sensor surface can be measured. It is important that the sensor surface is functionalized according to the type of molecule being detected. The magnetoelastic sensor will be more precise if it measures very low mass change. Therefore, enlarging the sensor surface area makes a significant contribution to the sensitivity of the sensor. In this context, TiO_2_ nanotubes (TiO_2_-NTs) was coated on the MES surface in order to increase the surface area of MES and the humidity sensing properties of the prepared MES are reported. Although there have been some studies regarding the humidity sensing properties of TiO_2_-coated MES, in this study, we use TiO_2_ in its nanotube form for the first time and we present detailed results compared to previous studies.

## 2. Experimental

### 2.1. Synthesis of TiO_2_-NTs

The synthesis of TiO_2_-NTs was performed using a hydrothermal process. For this purpose, a TiO_2_ nanopowder was synthesized by a reflux method. Titanium isopropoxide Ti(OPri)_4_ (97%, purchased from Alpha) as a precursor was dissolved in 2-propanol (99.5%, Sigma Aldrich, MI, USA) for 2-PrOH/Ti(OPri)_4_:10 (n/n) and stirred for 15 min at 800 rpm at ambient temperature to form solution A. It contains mol/mol ratios of H_2_O/Ti(OPr^i^)_4_ n/n = 10 and H_2_SO_4_/Ti(OPr^i^)_4_ n/n = 0.01 were mixed homogenously by magnetic stirring and allowed to stand on a magnetic stirrer at 800 rpm for 30 min to form solution B. Solution A was added to solution B dropwise with continuous stirring at 800 rpm at ambient temperature for 30 min to ensure the formation of a homogeneous solution. The mixture was transferred to reflux at 110 °C for 6 h to complete the synthesized TiO_2_-NPs. Centrifugation was carried out to remove the solvents from the medium and dried at 80 °C in a vacuum oven for 8 h to achieve the anatase phase of titania. TiO_2_-NPs (2 g) were dispersed in 40 mL of 10 M NaOH ultrasonically, followed by hydrothermal treatment at 180 °C for 48 h in a Teflon-lined autoclave. The solid phase was then washed with 0.1 M HCl and distilled water at pH 6 to 7 and the subsequently filtered TiO_2_-NTs were dried at 80 °C for 8 h. 

### 2.2. Measurement Systems

**Figure 1:**
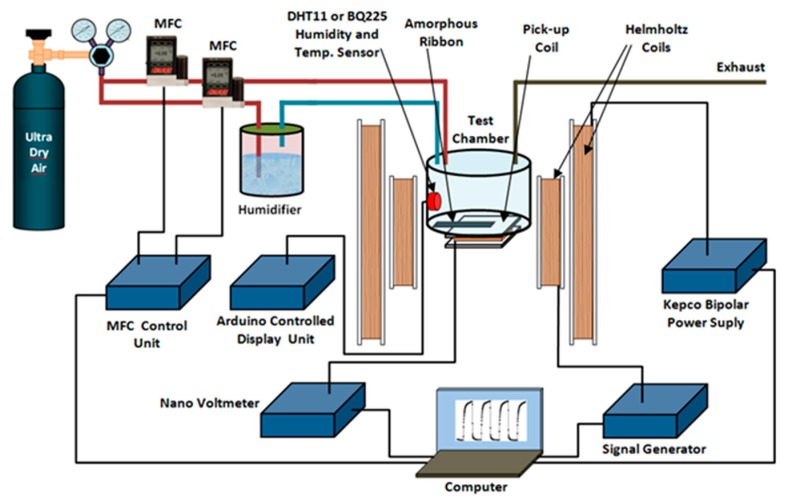
Schematic illustration of humidity measurement system.

The block diagram of the humidity measurement system is shown in Figure 1. The MES was placed into the bottom section of the humidity sensing unit and the MES was free to vibrate, no clamping was applied to the sample during the measurements. The pickup coil was placed directly below the sample and the distance between the magnetoelastic sensor and pickup coil is about 4 mm, but outside the humidity measuring unit. The wire gauge for the pickup coil is 37 A. ~10 A/m AC magnetic field was applied to the sample using a signal generator and the frequency of the signal was varied in the desired range using the measurement software. The AC magnetic field leads to the vibration of the ribbon longitudinally and induces voltages in the pickup coil. To minimize external noise signals, the pickup coil was placed parallel to the direction of the magnetic field, so that the signal generated by the AC magnetic field applied in the coil is minimal and the signal generated in the coil predominantly comes from the sample. The pickup coil has dimensions of 4 cm × 4 cm and 500 turns of copper wire. Two Helmholtz coils were used in the magnetoelastic measurements system, the Helmholtz coil used to generate DC bias magnetic field has a diameter of 52 cm and Helmholtz coil used to produce AC magnetic field has a diameter of 32.5 cm. 

A temperature sensor was placed into this unit and the temperature was monitored during the humidity measurements and no major temperature variation was observed during the testing. The humidity of the measuring unit was controlled using two flow meters, one flow meter controlling the dry air flow and the other humidified air, so that two humid and moisture-free gases were supplied to the measuring system at the desired rate and different humidity values were generated in the measuring system, the total flow rate usually kept constant to be around 400 sccm. The resonant frequency, frequency shift, and the mass change on the MES were measured using the homemade system. The measurement system can measure resonance frequency with 1 Hz sensitivity. All parameters during the measurements were controlled by a computer. 

The crystal phase of the TiO_2_-NTs was analyzed by X-ray diffraction (XRD) using a Rigaku RadB-DMAX II diffractometer with Cu Kα radiation (λ = 1.5418 A) in the region 2θ = 25.3°. For a description of the surface morphology, a Leo Evo 40 model scanning electron microscope (SEM) was used.

### 2.3. Magnetoelastic Sensor Preparation

2826MB (Fe_40_Ni_38_Mo_4_B_18_) amorphous ferromagnetic ribbons was used as MES. The ribbons were cut with 30 mm long and 4 mm wide using a dice saw. All samples were cleaned in an ultrasonic bath with acetone and deionized ultrapure water. Afterwards, the ribbons were quickly dried. Then, a Cr film of 100 nm thickness and Au film of 100 nm thickness were coated on both sides of the ribbon using a thermal evaporator Vaksis-midas PVD/4T thin film system. Coated ribbons were then heated at 200 °C for 3 h to ensure good adhesion of gold. A thin layer of TiO_2_-NTs on the surface of the Cr-Au plated ribbons was formed using a drop-casting method. About 20 µL of TiO_2_-NTs in ethanol were dripped onto the surface of the plated ribbon using a micropipette. Ethanol was then evaporated and later, it was observed that TiO_2_-NTs were clustered on the surface of the plated ribbon, thus MES is prepared as a humidity sensor.

In this study, four different MES with various TiO_2_ coating masses were prepared and the effect of the amount of coated TiO_2_ was also investigated with 0.26, 0.96, 1.75, and 2.64 µg of TiO_2_ coated to give MES-1, MES-2, MES-3, and MES-4, respectively.

## 3. Results and Discussion

**Figure 2:**
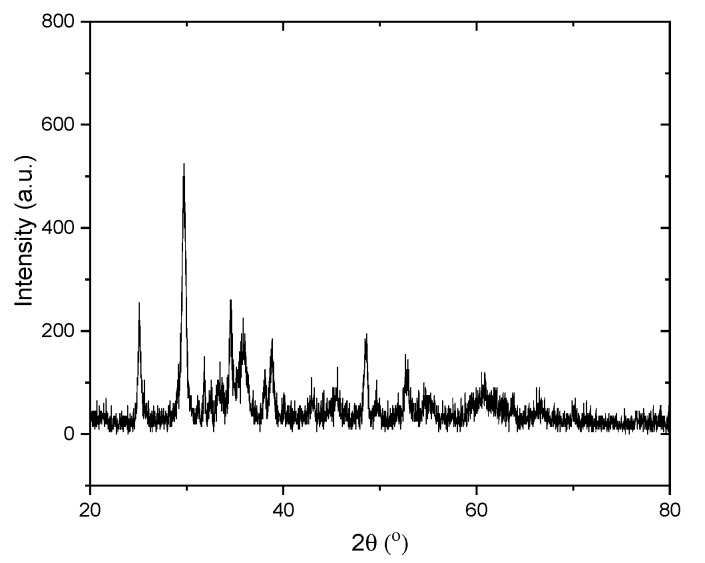
XRD patterns of the TiO_2_ nanotubes (TiO_2_-NTs).

**Figure 3:**
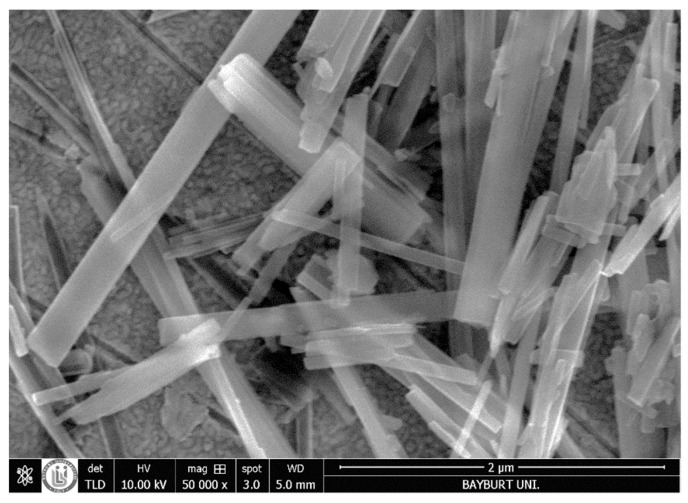
SEM image of TiO_2_-NTs.

The XRD pattern of the TiO_2_-NTs is given in Figure 2. It was identified that the diffraction peaks at 2θ = 25.24, 37.62, 48.22, and 54.72° are ascribed to the anatase phase; however, the 2θ = 30° highest peak belongs to the brookite phase or NaO_3_. Similar results were reported by Arruda et al. [35]. The SEM images of the TiO_2_-NTs are presented in Figure 3. The image shows clusters of TiO_2_-NTs. Most of the samples have a translucent appearance. Therefore, the samples below one of the upper samples are mostly visible. This is important to demonstrate that the samples are mostly tubular and a small amount of TiO_2_ with a solid wire shape also exists in the produced samples. This can be related to the production process, in other words, the dissolution-recrystallisation is always involved in the hydrothermal process and the products include NTs or nanowires, as reported in earlier studies [25].

**Figure 4:**
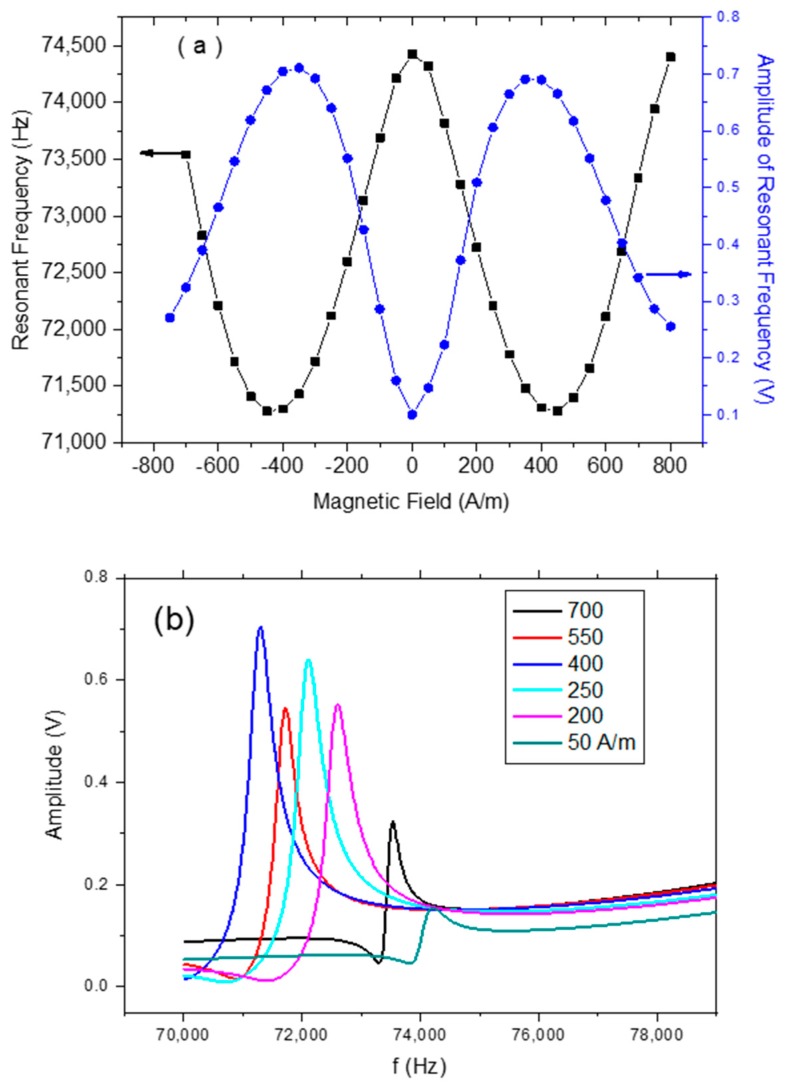
(**a**) Magnetic field dependence of resonant frequency and vibration amplitude of magnetoelastic sensors (MES)-1. (**b**) Frequency spectrum of MES-1 at various magnetic fields.

Figure 4a shows the variation of resonant frequency as a function of applied DC magnetic field at room temperature. It can be seen that resonance frequency first decreases and then increases with applied magnetic field, which agrees with previously reported results. Figure 4b shows the frequency response of MES without any coating at various magnetic fields. The vibration of amplitude reaches a maximum around the anisotropy field, we therefore used a 400 A/m DC bias magnetic field in all the humidity measurements.

**Figure 5:**
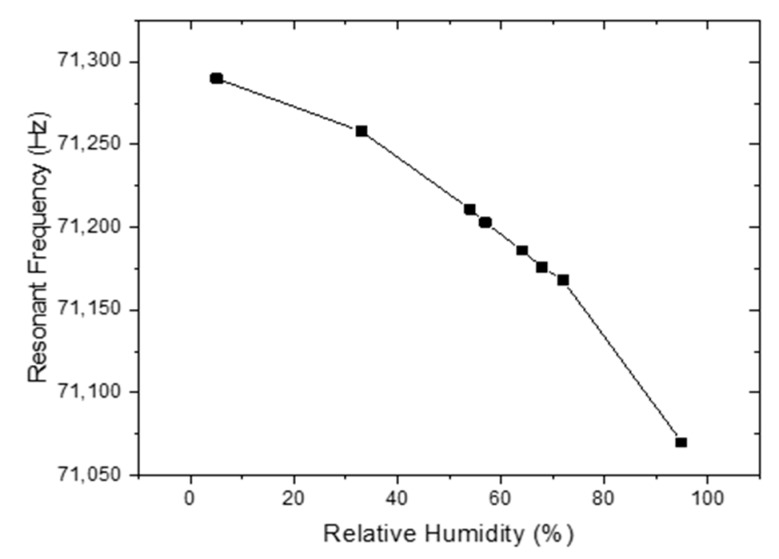
Variation of resonant frequency of MES-1 as a function of relative humidity levels.

The TiO_2_-NT deposited MES were calibrated using various salts and BQ225 and DHT11 humidity sensors. It is well known that saturated LiCl, MgCl_2_, K_2_CO_3_, Mg(NO_3_)_2_, NaCl, KCl, and K_2_SO_4_ solutions have RH levels of 11%, 33%, 43%, 52%, 75%, 85%, and 97%, respectively. The different humidity levels using these salts were applied to BQ225 and DHT11, the humidity sensor and calibrations of these sensors were checked. Then, we have used BQ225 and DHT11 sensors to monitor the humidity level in the test chamber, so the variation of resonant frequency of TiO_2_-NT deposited MES as a function of humidity level was obtained (Figure 5).

**Figure 6:**
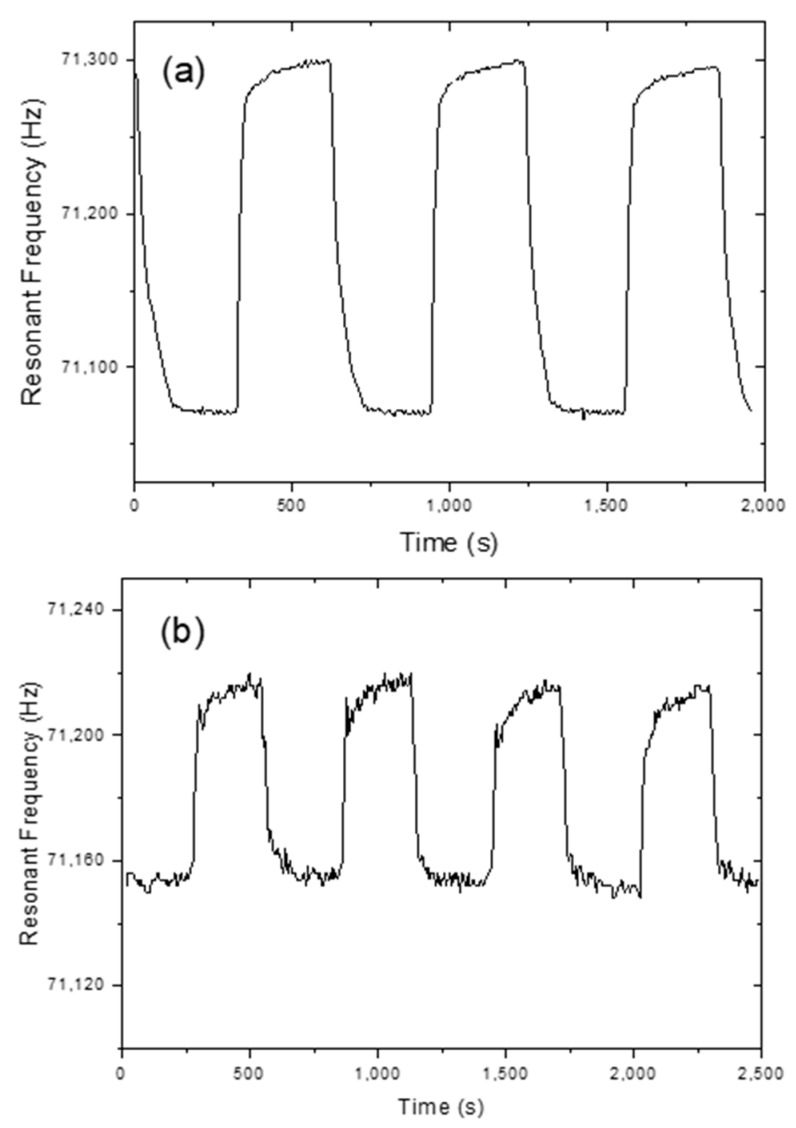
Frequency response of TiO_2_ NT-coated MES-1, (**a**) for cycling between 5% and 95% RH, (**b**) for cycling between 50% and 75% RH.

All measurements were performed at ~26 °C. The humidification and dehumidification response time measured with the TiO_2_-NT coated MES-1 was recorded as follows: First 5% RH then 95% RH was applied for some time and the cycle was thus continued. In addition, with the same sensor, 50% and 75% RH were cycled (Figure 6). The sensor reacts very quickly in both cases. At the end of each cycle, the sensor output reaches the same value, indicating that the sensor hysteresis is too small. 

**Figure 7:**
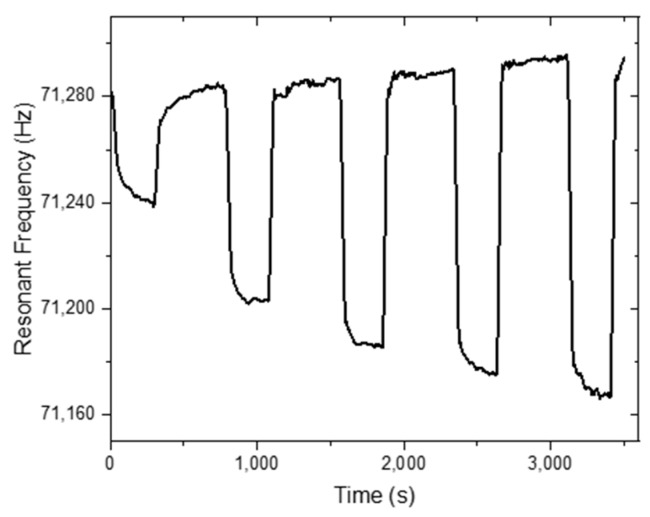
The static humidity response of the MES-1 for 41%, 58%, 63%, 69%, and 73% RH cycles, respectively.

**Figure 8:**
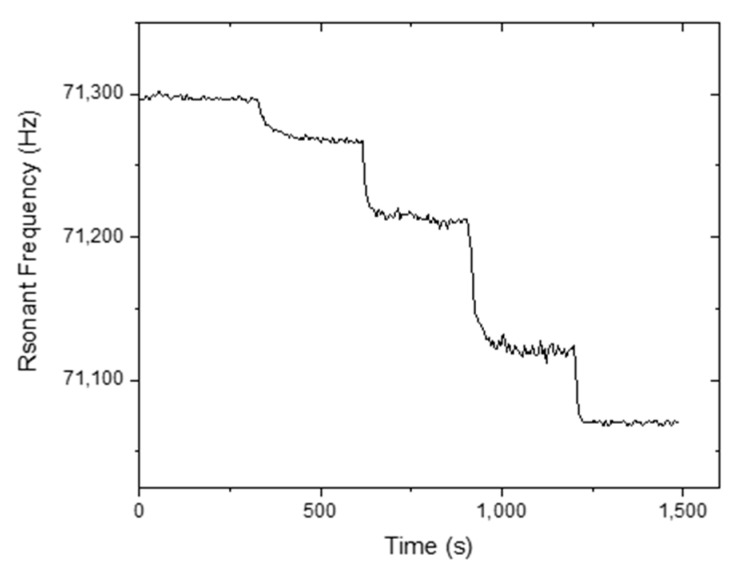
Frequency response of MES-1 for 5%, 25%, 54%, 85%, and 95% RH humidity levels, levels were applied step by step.

**Figure 9:**
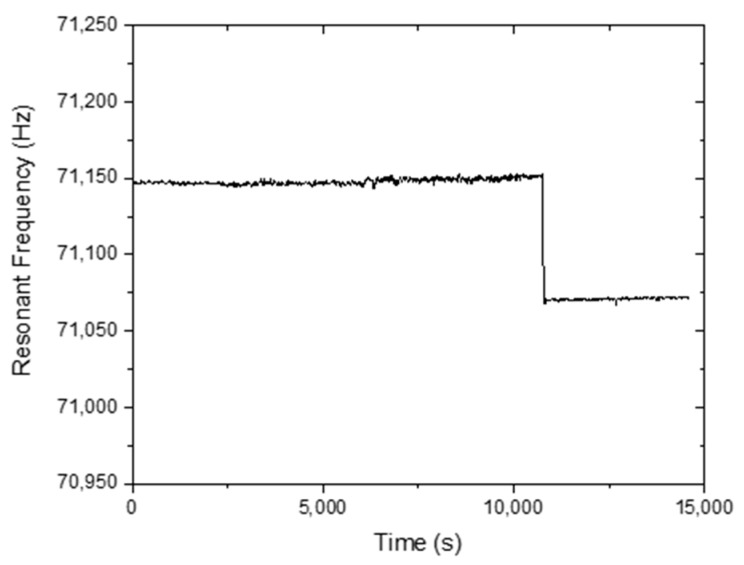
Long-term frequency variation of MES-1 at two different 77.5% and 95% RH levels.

The static humidity detection response of the MES-1 is shown in Figure 7 and Figure 8. The humidity value was first adjusted to 5% and then to 41%, 58%, 63%, 69%, and 73%, respectively, and between each step, the humidity value was brought to 5% (Figure 7). In contrast, in Figure 8, different humidity values, including 5%, 25%, 54%, 85%, and 95% RH, were applied step by step. Figure 7 and Figure 8 show that a very small change in the humidity values can be easily detected. Figure 9 shows the long-term stability of the TiO_2_-NT coated MES-1 measured at different 77.5% and 95% RH levels. The sensor was continuously tested for ~15,000 s at 4 s intervals. It was observed that the free frequency response of the TiO_2_-NT coated MES was almost unchanged.

**Figure 10:**
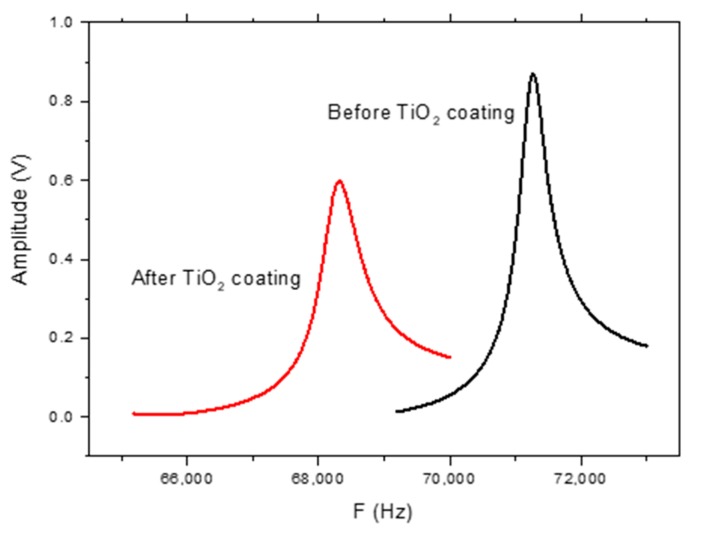
Frequency spectrum of MES-4, before and after TiO_2_-NTs coating at a 400 A/m magnetic field.

**Figure 11:**
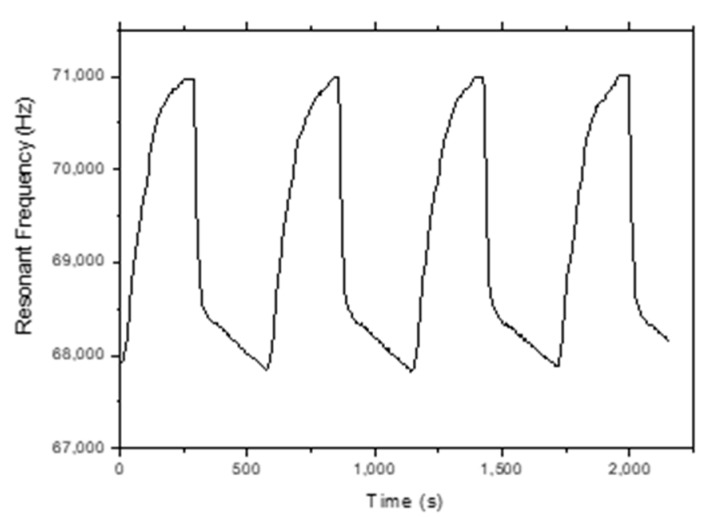
Frequency response of TiO_2_-NT-coated MES-4 for cycling between 5% and 95% RH.

**Figure 12:**
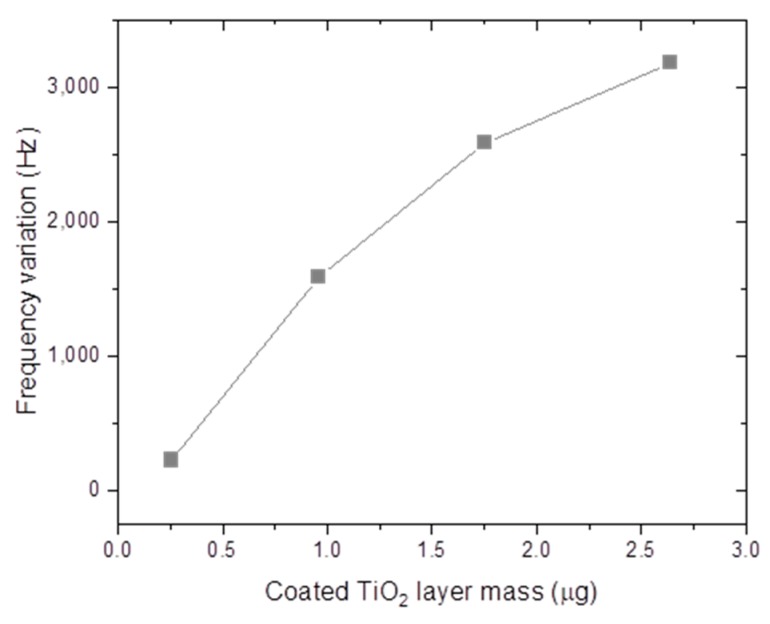
Effect of coating layer mass on the frequency response of MES.

Figure 10 shows the frequency response of the MES-4 sample for 5% and 95% RH levels at a 400 A/m DC magnetic field. In addition, the humidification and dehumidification response times were measured for the TiO_2_-NT-coated MES-1, MES-2, MES-3 and MES-4 for 5–95% RH cycles. Such a cycle for the MES-4 sample is shown in Figure 11. A ~3180 Hz variation in resonant frequency of the MES-4 was observed. Same measurements were also performed for MES-2 and MES-3, the frequency changes are 1590 and 2584 Hz, respectively. The results are shown in Figure 12, it can be seen that resonant frequency change for 5–95% RH cycles increases with increasing mass of the coated TiO_2_-NTs.

Magnetoelastic humidity sensors have been studied by Jain et al. [19]. A thin layer of Al_2_O_3_ was coated onto a 2826MB amorphous ribbon, and they tested the sensor at two different humidity levels, 2% and 98%. They found that the sensor shows a 500 Hz change in the resonance frequency for these humidity changes, and it takes approximately 2 h to obtain a steady-state response. Craig et al. [17,18] also coated a porous honeycomb structure of TiO_2_ onto 2826MB amorphous alloys, and they found that stabilizing the sensor output for 60% and 2% RH level cycling takes about 30 min. In this study, the stabilization of MES-1 sensor output for 95% and 5% RH level cycling takes about 40 s, as it can be seen that the sensor response time in this work is much faster than that of previously reported magnetoelastic humidity sensors. However, it was also observed that the sensor response time increases with the increasing coated mass of TiO_2_. It should be noted that there are differences between previous studies [17,18] and this study; previously, the testing chamber was relatively large compared to the humidity chamber used in this study, and the mass of the coated layer was also very high.

It has been reported that a hollow ball-like TiO_2_ film coated QCM sensor exhibited excellent humidity sensing performance in terms of higher sensitivity and shorter response/recovery time compared with the nanosphere and nanoflower TiO_2_ nanostructure [36] since, a hollow structure has a higher specific surface area, which brings much more active sites (surface defects and oxygen vacancies) for water molecule adsorption [37]. Moreover, the porous structure was beneficial to make water molecules diffuse quickly, which can make the sensor display shorter response and recovery times [38]. We assumed that this might be the reason a slightly better humidity response was obtained compared to previously reported magnetoelastic humidity sensors. 

A number of methods have been developed for humidity detection, including capacitance [39,40,41,42], resistance [14], optic [43], microwave [44], magnetoelastic [17,18,19], surface acoustic wave [45], and quartz crystal microbalance (QCM) techniques [46,47]. In this study, a magnetoelastic sensor was used to detect humidity. Response and recovery times were measured to be 40 and 60 s for the MES-1 sensor, respectively. This is relatively high compared to some humidity sensors [36,46,48,49,50], but the MES-1 sensor still shows better response and recovery time compared to some other types of humidity sensors [51,52]. Moreover, in the previous studies, humidity sensor sensitivities have been reported to be 29.1 [53], 0.54 [54], 41.1 [55], 9.4 [56], and 23.1 Hz/%RH [57]. The MES-4 sensor sensitivity of this work was about 35.3 Hz/%RH, so this indicates that the MES-4 sensor shows very good sensitivity compared with the results of some previous studies.

## 4. Conclusions

In conclusion, a TiO_2_-NT film on Fe_40_Ni_38_Mo_4_B_18_ amorphous ferromagnetic ribbons was successfully fabricated using a drop-casting method. The effect of coated layer mass was reported for the first time for magnetoelastic sensor and different amounts of TiO_2_-NTs were coated on the amorphous ribbon and it was found that the resonant frequency change for 5–95% RH cycles increases with increasing mass of the coated TiO_2_-NTs. Furthermore, it has been found that these increases in the low coated layer mass are sharp and then this sharpness decreases. For the sample coated 2.64 µg of TiO_2_-NTs, a ~3180 Hz change in the resonant frequency was observed for variation humidity levels of 5–95% RH. The humidity sensing properties of the TiO_2_-NT film on MES is very sensitive to humidity changes and reversible adsorption/desorption performance, which is indicative of a good humidity sensor.
